# Understanding Alkali Contamination in Colloidal Nanomaterials to Unlock Grain Boundary Impurity Engineering

**DOI:** 10.1021/jacs.1c11680

**Published:** 2022-01-04

**Authors:** Se-Ho Kim, Su-Hyun Yoo, Poulami Chakraborty, Jiwon Jeong, Joohyun Lim, Ayman A. El-Zoka, Xuyang Zhou, Leigh T. Stephenson, Tilmann Hickel, Jörg Neugebauer, Christina Scheu, Mira Todorova, Baptiste Gault

**Affiliations:** † Max-Planck-Institut für Eisenforschung GmbH, Max-Planck-Straße 1, 40237 Düsseldorf, Germany; ‡ Department of Materials, Royal School of Mines, Imperial College, London SW7 2AZ, United Kingdom

## Abstract

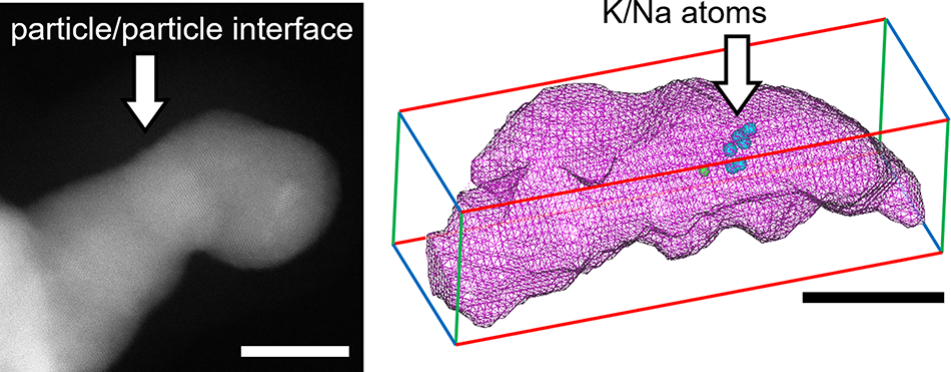

Metal nanogels combine a large surface area, a high structural stability, and a high catalytic activity toward a variety of chemical reactions. Their performance is underpinned by the atomic-level distribution of their constituents, yet analyzing their subnanoscale structure and composition to guide property optimization remains extremely challenging. Here, we synthesized Pd nanogels using a conventional wet chemistry route, and a near-atomic-scale analysis reveals that impurities from the reactants (Na and K) are integrated into the grain boundaries of the poly crystalline gel, typically loci of high catalytic activity. We demonstrate that the level of impurities is controlled by the reaction condition. Based on *ab initio* calculations, we provide a detailed mechanism to explain how surface-bound impurities become trapped at grain boundaries that form as the particles coalesce during synthesis, possibly facilitating their decohesion. If controlled, impurity integration into grain boundaries may offer opportunities for designing new nanogels.

## Introduction

Despite over 150 years of the “wet” chemical synthesis of colloidal metal nanoparticles and other nanostructures, many aspects of their structure and composition remain elusive, leading to Xia et al. saying “an art rather than a science”.^1^ The wet-synthesis of nanostructures typically involves adding a reducing agent to a solution containing a metal precursor. Due to its excellent reducing properties,^2^ NaBH_4_ is the most commonly used agent in laboratory and industrial applications,^3^ the so-called NaBH_4_ reduction method.^4^ Early nuclei of nanocrystals form and are grown by the agglomeration of the reduced metal atoms, becoming stable nanocrystals upon reaching a critical size.^5^ Yet, the interaction of alkalis (Na or K) with the growing nanocrystals is rarely considered in growth models.

The rapid generation of metal atoms following the introduction of a strong reducing agent results in a high concentration of crystal nuclei, which eventually coalesce into a nanogel and become a metal nanoaerogel (MNA) structure.^6,7^ MNAs are an emerging class of self-supported porous materials with potential in electrocatalysis, surpassing commercial metal-based catalysts because of their structural stability and efficient mass and electron transfer channels.^8,9^ MNAs have been extensively studied across an array of catalytic applications such as the oxygen reduction reaction,^10,11^ the glucose oxidation reaction,^12,13^ and the ethanol oxidation reaction.^14,15^

MNAs synthesized by the NaBH_4_ reduction of a metal precursor are often perceived as purely metallic, *i.e.*, without impurities integrated in their complex nanoporous structure.^16,17^ Impurities inside MNAs can modify their stability and electronic structure and hence their reactivity. Here, we study the controlled integration of impurities into nanogels by combining microscopy and microanalysis at the near-atomic scale,^18,19^ with *ab initio* density functional theory (DFT) calculations to investigate the energetics and incorporation of impurities into the nanostructures, in particular at crystalline defects. The mechanism we outline will facilitate tailoring MNAs for specific catalytic reactions using insights from atomistic simulations.^20^

## Results and Discussion

**Figure 1:**
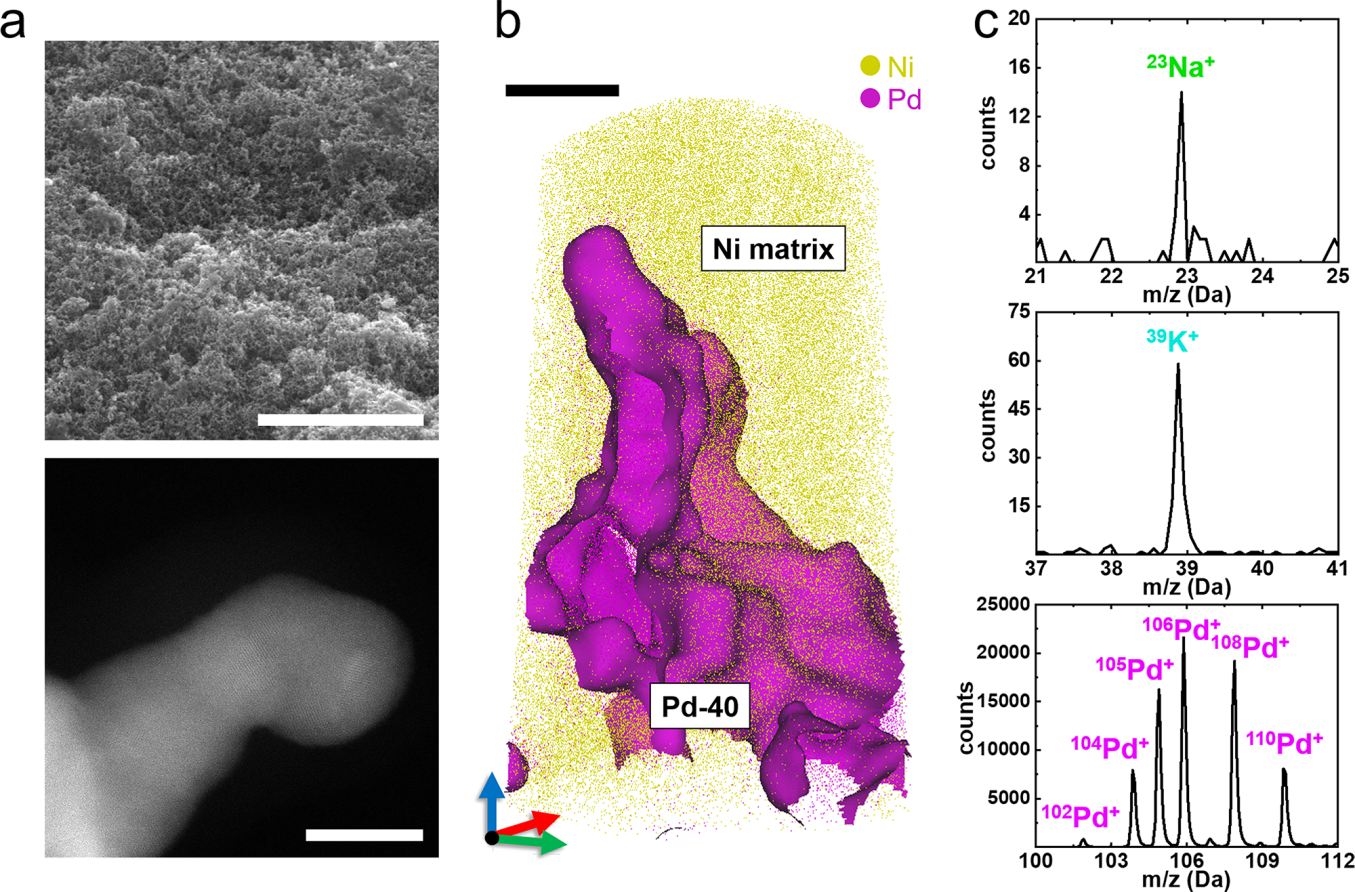
Characterization of Pd-40 nanogels. (a) SEM and HAADF-STEM images of as-synthesized Pd-40 gels. Scale bars are 5 μm and 10 nm for the SEM and STEM images, respectively. (b) 3D atom map of Pd-40 gels embedded in a Ni matrix (scale bar is 10 nm). A one-dimensional composition profile positioned perpendicular to the matrix–MNA interface is shown in Figure S3. (c) Illustration of major and minor peaks in the following three different mass-to-charge ratio ranges: Na, K, and Pd. The overall mass spectrum is presented in Figure S4.

We synthesized two Pd-gels with two mole ratios of the NaBH_4_ reductant to the Pd-precursor (R/P), namely, 40 and 0.1, that are referred to as Pd-40 and Pd-0.1, respectively (for details, please see the Experimental Section). After synthesis, the gels were thoroughly washed three times with distilled water to remove surface residuals. Figure 1a displays micrographs of the as-synthesized Pd-40 obtained by scanning electron microscopy (SEM) and high-angle annular dark field-scanning transmission electron microscopy (HAADF-STEM). The three-dimensional (3D) highly complex network of pores of the MNA structures is readily apparent, with an average ligament size of approximately 15 nm. Energy-dispersive X-ray spectroscopy (EDS) (Figures S1 and S2) hints at the presence of Na and K. The complex geometry of the specimen and the low concentration of the impurity elements made the combined quantification and highly spatially resolved localization within the MNA extremely challenging.^21,22^

We then performed atom probe tomography (APT) using the protocol outlined in ref (23) and embedded the MNA in a Ni matrix (see the Experimental Section). The 3D atom map for the Pd-40 embedded in Ni is shown in Figure 1b. An isocomposition surface of 50 at. % Pd highlights the complex morphology of Pd-40, which is compatible with the STEM imaging. The APT mass spectrum from the Pd-rich region, shown in Figure 1c, contains peaks associated with Na and K at 23 and 39 Da, respectively, along with the isotope of singly charged Pd (102 to 110 Da). These impurities, *i.e.* Na and K, likely originate from the reducing agent (NaBH_4_) and the Pd precursor (K_2_PdCl_4_), respectively. Although alkali metals are often believed to be surface residuals on the nanoparticle system, the Na and K were detected inside the Pd-40 network with compositions of 94 ± 2 and 512 ± 50 atomic parts per million (appm), respectively. Cl only weakly binds to Pd surfaces in aqueous solutions,^24^ and no Cl was detected.

To unveil the origin of these impurities, we studied the Pd-0.1 (R/P = 0.1), which had the same concentration of the Pd precursor but less NaBH_4_ in the solution. The morphology and ligament size are similar to those of Pd-40 (Figures S5 and S6). Du et al.^6^ reported an influence of the R/P ratio on the ligament size of Au MNAs with a low (<2) and high (>50) R/P. Here, the Pd precursor solution is more highly concentrated (0.01 versus 0.0002 M), leading to a higher concentration of nuclei to form the MNA. Pd-0.1 contains a similar amount of K (512 versus 642 appm) in both MNAs, and its synthesis involved the same concentration of the K_2_PdCl_4_ precursor. In contrast, the lower concentration of the Na-containing reductant in solution leads to a lower incorporation of Na (22 versus 94 appm). Table S1 reports the compositions of both samples.

A new batch with a one-to-one mole ratio, Pd-1, was also synthesized. Figure S7 shows the 3D atom maps and sectioned tomogram of the Pd-1 sample. Inside the gel structure, Na and K were detected at 73 ± 7 and 417 ± 16 appm levels, respectively. A similar amount of K was detected compared to that where the same concentration of the precursor was involved, whereas the Na content was between that of Pd-0.1 and Pd-40 samples (see Figure S8).

**Figure 2:**
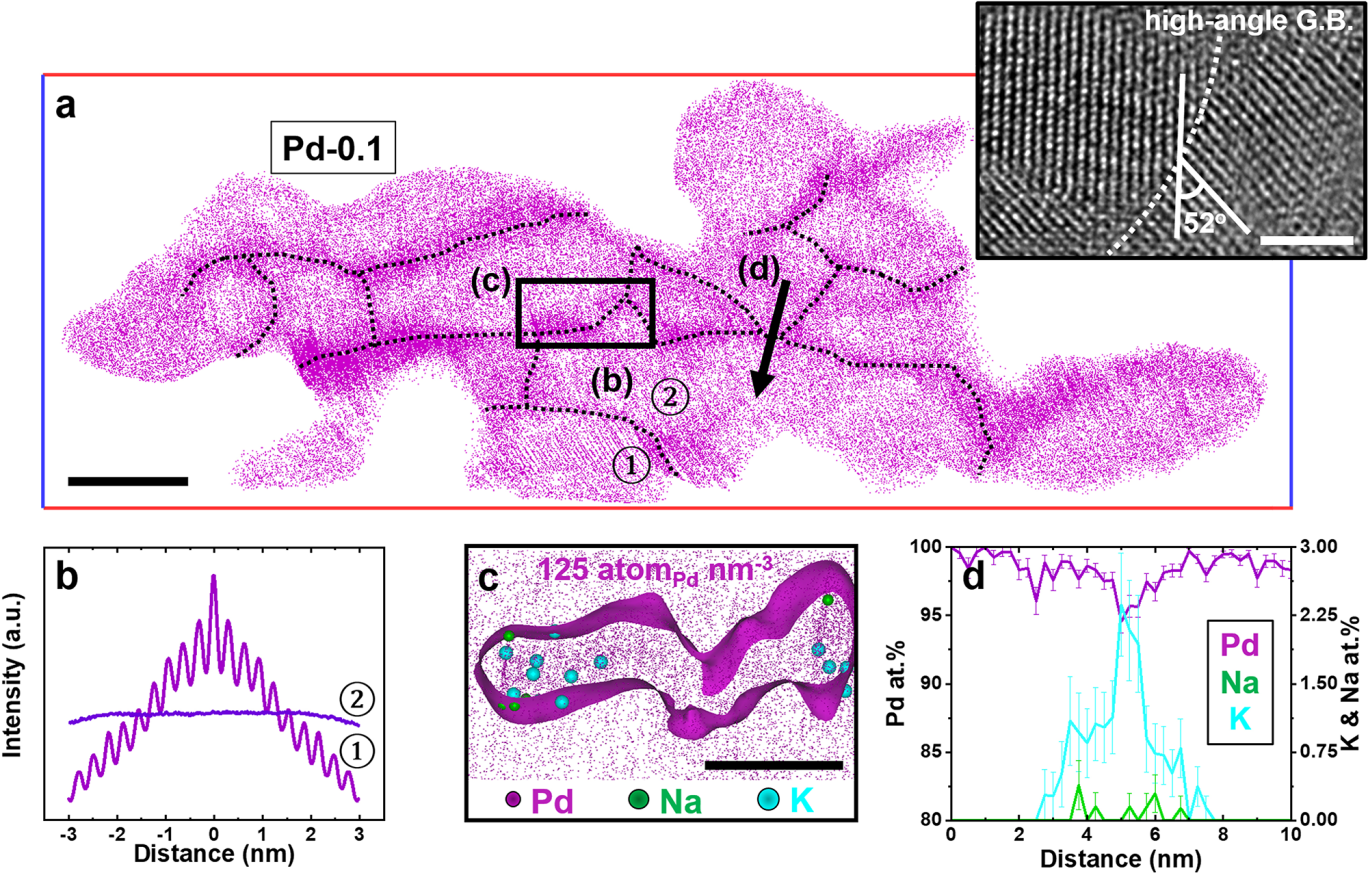
Grain boundary studies for Pd-0.1 nanogels. (a) A 1 nm thin-sliced tomogram from a 3D atom map (Figure S5) of Pd-0.1 gels (iso-composition surface >90 at. % Pd). The scale bar is 10 nm. The dotted black line represents grain boundary features. The inset shows a high-resolution TEM image of Pd-0.1 (scale bar is 2 nm). (b) Spatial distribution maps along the *z*-axis from the grains of two different nanogels. (c) Extracted grain boundary tomogram with the isodensity surface of 125 Pd atoms/nm^3^. The scale bar is 5 nm. (d) 1D compositional profiles of detected Na, K, and Pd elements.

During the MNA synthesis, the crystals start to coalesce^25^ and merge with each other with no particular crystallographic relationship following the initial growth of nuclei reaching a critical size, conversely to an orientated attachment.^26^ Therefore, MNAs are agglomerations of randomly oriented crystal grains with numerous interfaces, *i.e*., grain boundaries (GBs).^27^ A 1 nm thick slice through the APT reconstruction, shown in Figure 2a, reveals the presence of such GBs in the Pd MNA. The apparent higher point density at the junction nanocrystals is related to aberrations in the ion trajectories.^28^ GBs are marked by black dotted lines. In Figure S9 and the inset in Figure 2a, the high-resolution (HR-) TEM image shows multiple high-angle grain boundaries (>15°) within the poly crystalline nature of Pd nanogels. In addition, grain ① shows a periodic arrangement of atoms pertaining to a set of crystallographic planes in the APT data, as revealed by the so-called spatial distribution map^29^ plotted in Figure 2b, which terminates at the junction with the neighboring grain ②. Therefore, each grain in Pd-0.1 has a different orientation. Na and K are mostly found along the GBs, as shown in Figure 2c, and a composition profile calculated along the black arrow (Figure 2d) indicates that the GB contains 0.5 at. % Na and 2.3 at. % K. The segregation to interfaces is often reported in bulk materials^30,31^ but has not been studied in MNAs.

**Figure 3:**
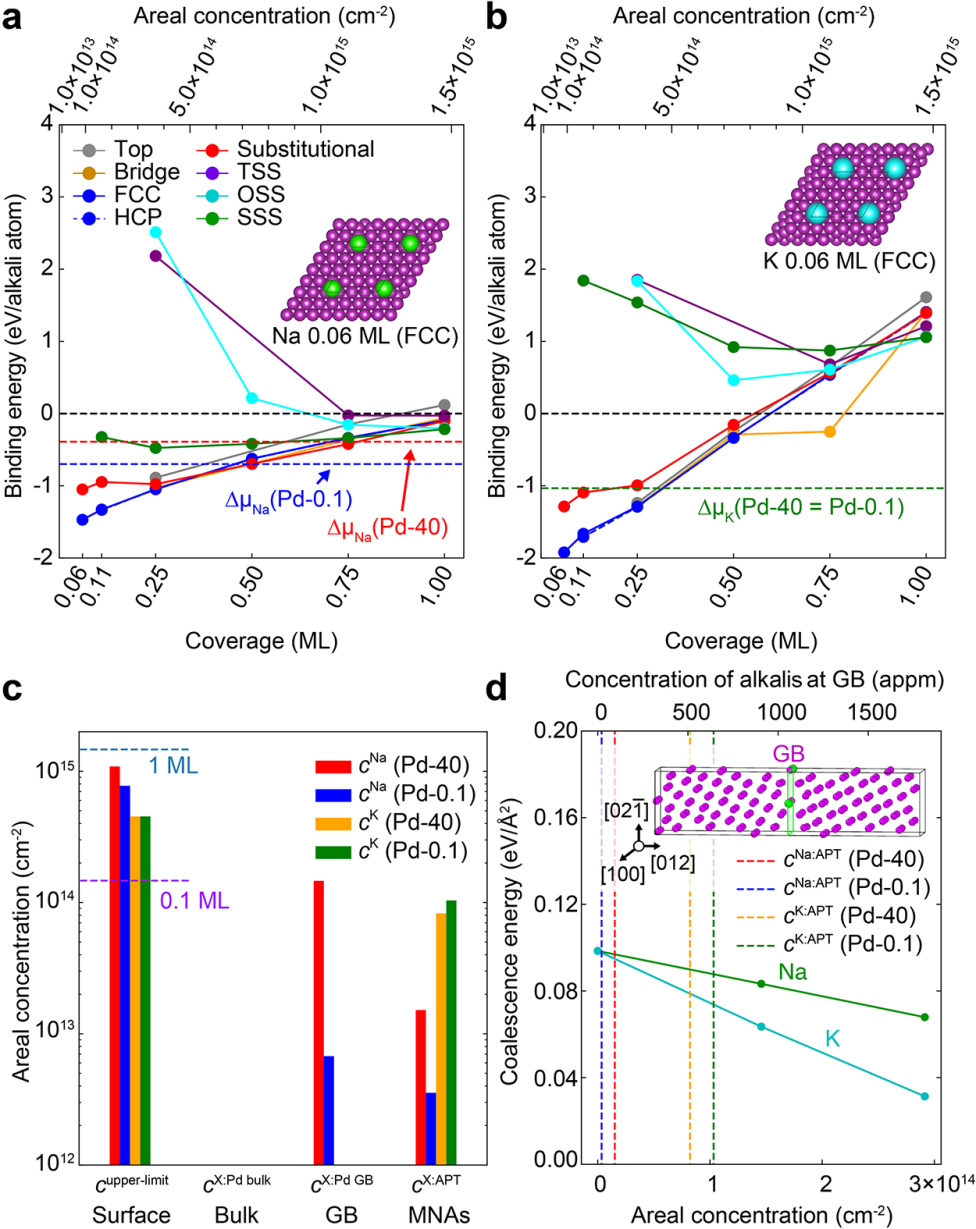
Surface adsorption and grain boundary energy calculations. The binding energies with respect to the alkali BCC bulk phase (*E*_b_) of (a) Na and (b) K adsorbates at the Pd (111) surface and subsurface for several adsorbate coverages in the range 0.06–1 ML. Solid lines of different colors correspond to different binding sites for the alkali atom. Colored horizontal dashed lines show the shifts in the chemical potentials corresponding to alkali ions in solution for the given experimental conditions with respect to the alkali BCC bulk phase. Insets show examples of binding at FCC sites for 0.06 ML of Na (green) and K (cyan) on the *p*(2 × 2) Pd (purple) surface. (c) Plotted in a log scale are the thermodynamic equilibrium concentrations of alkalis on the surface at 300 K, in the bulk, and in the GB in addition to the experimentally measured concentrations of alkalis in MNAs for the considered experimental conditions. Horizontal dashed lines indicating areal concentrations of 1 and 0.1 ML surface coverages are shown as guides. (d) The coalescence energy of the GB is plotted as a function of the areal concentration of alkalis in the GB (assuming the concentration of alkalis in the GB to be identical to that on the surface), with the experimentally observed alkali contents shown as vertical dashed lines. The inset shows the supercell containing the ∑5 (012) Pd grain boundary used in the coalescence energy calculations.

To rationalize the impurity incorporation into the MNAs, we first evaluate the binding energy (*E*_b_) of Na and K adsorbates relative to their respective BCC bulk phase (zero reference for the chemical potential) at different binding sites on a Pd (111) surface using density functional theory (DFT). The modeled Pd surface is a proxy for a crystal nucleus, *i.e*., before coalescence. The threefold hollow sites (*i.e.*, FCC and HCP) are the most favorable binding sites for both alkalis at low coverages (Θ ≤ 0.25), Figure 3a and b, and for both the surface binding energy monotonously reduces with increasing coverage. We used the Nernst equation to calculate the chemical potentials from the ion concentration in solution for two synthesis conditions, and they are shown as colored horizontal dashed lines in each pane of Figure 3. The difference between *E*_b_ and the corresponding chemical potential for low and high concentrations in solution (blue- and red-dashed lines, respectively) is the net binding energy, which allows us to directly determine the equilibrium concentration of the alkali atoms at the surface. Figure 3c indicates that the surface concentrations of both Na and K are large, at 0.31 ML for K and 0.74 to 0.52 ML for Na, when the Na concentration in solution is reduced from 0.4 to 0.001 M.

APT analysis reveals Na and K atoms predominantly at GBs and not at the surface, from which they were likely leached by rinsing with water. To rationalize the presence of alkalis at GBs within Pd-MNAs, we first consider two extreme scenarios: (i) all alkali atoms chemisorbed at the surface become trapped during coalescence and (ii) the alkalis at the GBs can achieve a thermodynamic equilibrium with those in solution. Since coalescence brings two surfaces together, the GB concentration for case (i) would be twice the surface concentration. Since the experimentally observed Na and K concentrations at GBs are significantly lower, at least a (partial) equilibration takes place.

With regards to the second scenario, we computed the GB concentrations of Na and K in equilibrium with the corresponding chemical potential in solution for a ∑5 (210) [001] GB with a misorientation of 53.13° and an open structure using DFT (Figure 3c and the Supporting Information). Assuming that this ∑5 GB can serve as a proxy for a random high-angle GB, the computed equilibrium GB concentration of Na agrees qualitatively with experimental concentrations. For K, however, the measured concentration is orders of magnitude larger than predicted (1.0 × 10^14^ versus 4.2 × 10^–12^ cm^–2^). The high concentration of K atoms initially present at the surfaces is only partially released back in solution to achieve thermodynamic equilibrium during coalescence, and a substantial number of K atoms are kinetically trapped in the GB plane (roughly 20% for Pd-40 and 2% for Pd-0.1). The deduced sluggish kinetics of K compared to those of Na is also supported by a recent report for polycrystalline Mo and Nb, where the larger-sized K has a smaller diffusion coefficient than Na by a factor of 2–3.^32^

We then studied the GB coalescence energy, *i.e.*, the energy required to form a GB interface from two surfaces, as a function of the Na and K concentrations. Indeed, a large concentration of alkali atoms could energetically stabilize these metallic surfaces so much that forming a GB would be thermodynamically unfavorable and coalescence would be suppressed. As shown in Figure 3d, the coalescence energy is systematically positive and hence coalescence is energetically favorable. K almost halves the GB coalescence energy, whereas Na has relatively less influence.

From the perspective of the mechanical properties, the embrittlement of grain boundaries can result in the catastrophic facture of crystalline materials^30^ and would likely affect the lifetime and device durability of MNAs. In contrast, since the presence of K changes the energetics of GBs and hence their likelihood of formation, using an excess amount of K during synthesis could lead to an increase in the surface area-to-volume ratio and hence be beneficial for catalysis applications. We demonstrate this qualitatively in Figure S10, where STEM and APT analyes of a Pd-MNA synthesized with an excess of K from KCl show relatively thinner ligaments.

## Conclusions

**Figure 4:**
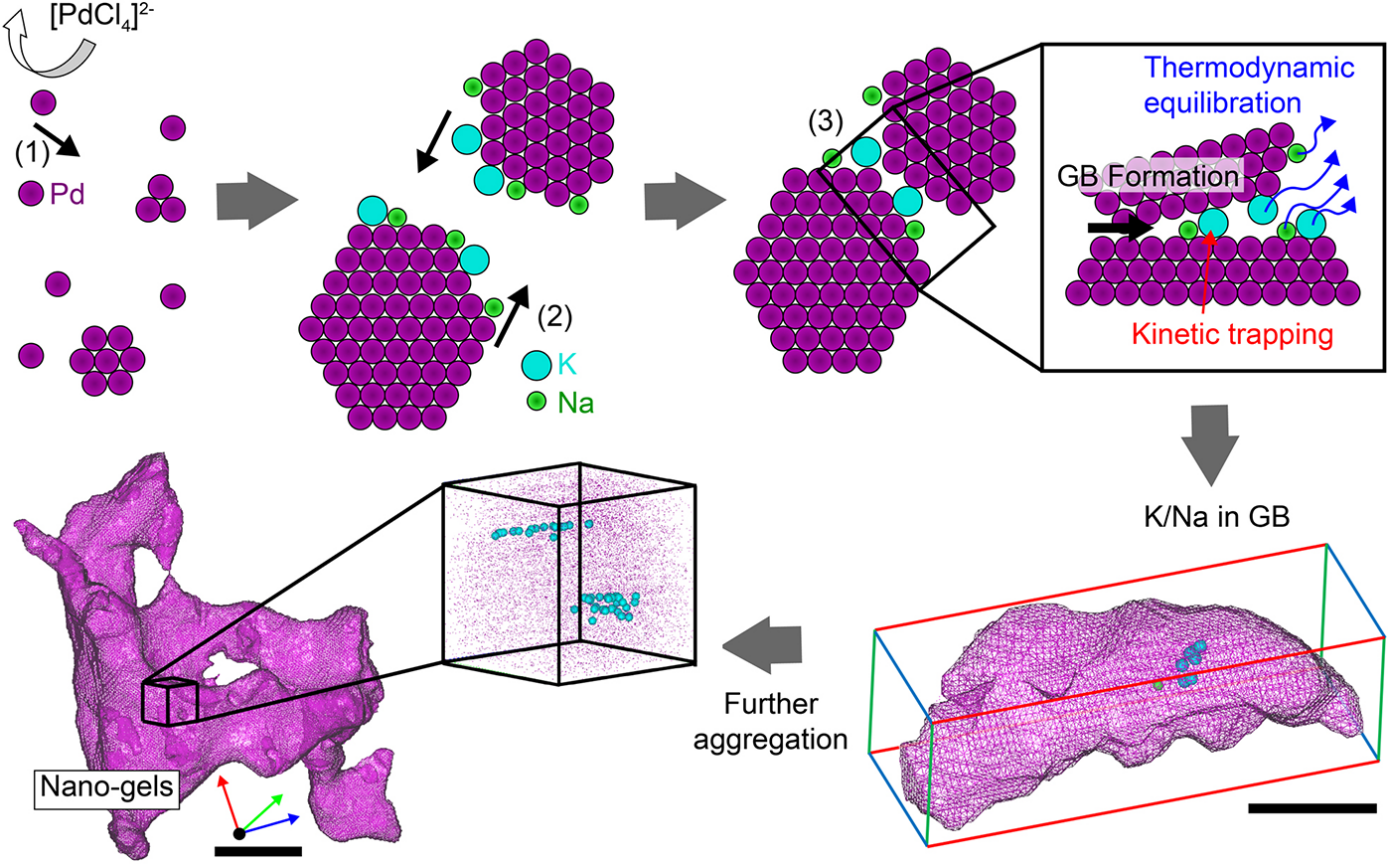
Alkali atom integration mechanism. Schematic illustration of steps comprising the formation mechanism of Pd nanogels, which are as follows: reduction of Pd atoms, formation of a Pd nuclei, coalescence of Pd nanocrystals, aggregation of primary Pd nanogels, and final Pd nanogels. The last two steps are from APT results (scale bars are 5 and 10 nm for the fourth and fifth steps, respectively). Purple wireframes represent the isocomposition surface of Pd at >90 at. %. Green and cyan dots represent Na and K atoms, respectively. During the nanogel formation, (1) the reduction of the Pd precursor to a Pd^o^ atom (purple), (2) the absorption of alkali atoms (K (cyan) and Na (green)), and (3) the coalescence of Pd nanocrystals with the integration of alkali atoms into the interface occur rapidly.

To conclude, our theoretical and experimental insights allow us to propose a general mechanism for alkali integration in nanostructures synthesized by wet chemistry, which is schematically illustrated in Figure 4. Nanocrystals form throughout the solution, with their metal–solution interface partly stabilized by impurities, *e.g*., K and Na. Through the agglomeration of these nanocrystals, a large number of impurities end up at the generated internal interfaces, *i.e*., grain boundaries. Impurities are then leached back in solution to reduce the gel’s internal energy. During this process, interfaces become increasingly more stable, but a substantial amount of the large-sized K atoms remain kinetically trapped by the moving surfaces of the growing nanocrystals.

Importantly, this opens the possibility of using grain boundary engineering (GBE) guided by information obtained *in silico* for the property optimization of colloidal nanomaterials. In oxide polycrystals, the influence of the space charge due to metal excess at the interfaces is well-studied. The dangling bonds of the excess metal in oxides will lead to bond electrons at the interface and will fill the electronic states near the conduction band, which lowers the material intrinsic band gap.^33−35^ GBE successfully allowed control over the mechanical and chemical properties of bulk metallic alloys (*e.g*., steel^36^ and Mg alloy^31^) and inorganic compounds (*e.g.*, CIGS^37^).

Our results show the potential in applying GBE to decorate GBs in freestanding nanomaterials. For example, the local change in the electronic properties at GBs in MNAs^38^ facilitates the adsorption of CO_2_ and provides fast kinetics for the CO_2_ reduction reaction.^39,40^ Properties could also be further enhanced by promoting the adsorption of specific elements selected from *ab initio* calculations. We also showed that the concentration of these impurities within the nanostructure is controlled by their initial concentration in solution and the relative energetics of the surface and GBs, providing levers to help future material design.

Eliminating the source of the alkali (*e.g*., NaBH_4_ reductant or a potassium-based metal precursor) in MNA synthesis adversely affects the high production rate; therefore, there is an inevitable inverse relation between the integration of impurities and efficient MNA production. In addition, impurities at the GB could reduce its cohesion, which is detrimental to the longevity of MNAs. Property optimization will hence depend on a subtle compromise, and the effective usage or removal of alkali atoms at GBs during coalescence appears to be pivotal for a successful application of GBE in MNA. There may also be opportunities to exploit impurity ingress to dope the material in order to obtain a strengthening effect to counteract the coalescence or act as a promotor to the catalytic activity.

## Experimental Section

### Synthesizing Pd Nanogels

For Pd-40 synthesis, 0.01 M K_2_PdCl_4_ (potassium tetrachloropalladate 99.99%, Sigma-Aldrich) was mixed with 0.4 M NaBH_4_ (sodium borohydride, 99.99%, Sigma-Aldrich); for Pd-0.1, 0.001 M NaBH_4_ was used. After the reaction completely stopped, a centrifuge was used to collect the Pd black powder, which was redispersed in distilled water. This process was carried out three times to remove excess residuals on the nanogels. Finally, the collected powder was then dried in a vacuum desiccator for a day.

### Sample Preparation for APT Measurement

As-synthesized Pd nanogels were prepared into a APT sample following the modified coelectrodeposition technique.^41,42^ For an electrolyte preparation, nickel(II) sulfate hexahydrate (98%, Sigma-Aldrich) and citric acid (99.5%, Sigma-Aldrich) were dissolved in 50 mL of distilled water. Here we used H-citric acid to avoid any possible Na or K introduction from the APT sample process. A constant current of −38 mA was applied for 1250 s to completely encapsulate colloidal Pd nanomaterials with Ni film.

### TEM Characterization

HAADF-STEM images were acquired using a JEM-2200FS TEM (JEOL) at 200 kV. Elemental mapping using EDS was carried out to investigate the chemical composition of the Pd nanogels. HRTEM images were obtained with an aberration-corrected FEI Titan Themis 60-300 microscope at 300 kV.

### APT Characterization

Pd gels or Ni APT specimens were prepared using the standard specimen preparation technique^43^ with a focused ion beam (FEI 600 DualBeam) and were subsequently were loaded inside a LEAP 5000 XS (CAMECA) system. The APT measurement was performed in pulsed laser mode at set temperature of 50 K. A detection rate of 1%, a laser pulse frequency of 200 kHz, and a laser energy of 60 pJ were used throughout the measurement. The acquired data set was then analyzed using IVAS 3.8.4 (CAMACA) software.

### Computational Calculation

The Vienna *AbInitio* Simulations Package (VASP) code^44^ employing the projector augmented wave (PAW) method^45^ was used for all DFT calculations. A plane-wave cutoff of 500 eV was used, which was sufficient to achieve a force convergence of 0.01 eV/Å and a total energy convergence of 10^–6^ eV. The generalized gradient approximation (GGA) due to Perdew, Burke, and Ernzerhof^46^ was used for the exchange-correlation approximation. Brillouin-zone integration was carried out using Methfessel–Paxton smearing. Γ-Centered *k*-point grids with the following *k*-points were used for Brillouin-zone integrations: (8 × 8 × 8) for the face-centered cubic Pd bulk, (8 × 8 × 1) for the Pd(111) *p*(1 × 1) surface unit cell, and (2 × 9 × 9) for grain boundary calculations. The *k*-point meshes in the surface calculations were equivalently folded according to the size of the considered surface cells. Electronic and ionic relaxations were carried out until the total energy convergence was less than 10^–5^ eV, respectively 10^–4^ eV per system. Within this setup, the obtained lattice parameter *a* = 3.959 Å and the cohesive energy *E*_coh_ = 3.63 eV of Pd fcc bulk agree well with previous theoretical^47,48^ and experimental^49^ results. Details on surface adsorptions in addition to grain boundary calculations and concentration analysis are presented in the SI.

## Supporting Information Available

Advanced characterization data, including the mass spectrum of the as-synthesized Pd sample, and details of the DFT calculation (PDF)
