# Supplementary material for: Understanding Alkali Contamination in Colloidal Nanomaterials to Unlock Grain Boundary Impurity Engineering
Source: J Am Chem Soc. 2022 Jan 4;144(2):987–94. doi: 10.1021/jacs.1c11680 (PMC8778649; doi:10.1021/jacs.1c11680)
Supplement: Supplementary file 1 — ja1c11680_si_001.pdf [file ja1c11680_si_001.pdf]

Supporting Information

Understanding alkali contamination in colloidal nanomaterials to unlock grain boundary impurity engineering

Se-Ho Kim[†,§,*], Su-Hyun Yoo[†,§,*], Poulami Chakraborty[†], Jiwon Jeong[†], Joohyun Lim[†], Ayman A. El-Zoka[†], Xuyang Zhou[†], Leigh T. Stephenson[†], Tilmann Hickel[†], Jörg Neugebauer[†], Christina Scheu[†], Mira Todorova[†], Baptiste Gault[†,‡,*]

[†]Max-Planck-Institut für Eisenforschung GmbH, Max-Planck-Straße 1, 40237 Düsseldorf, Germany

[‡]Department of Materials, Royal School of Mines, Imperial College, London, SW7 2AZ, United Kingdom

[§]these authors contributed equally

[*]corr. Authors: s.kim@mpie.de | yoo@mpie.de | b.gault@mpie.de

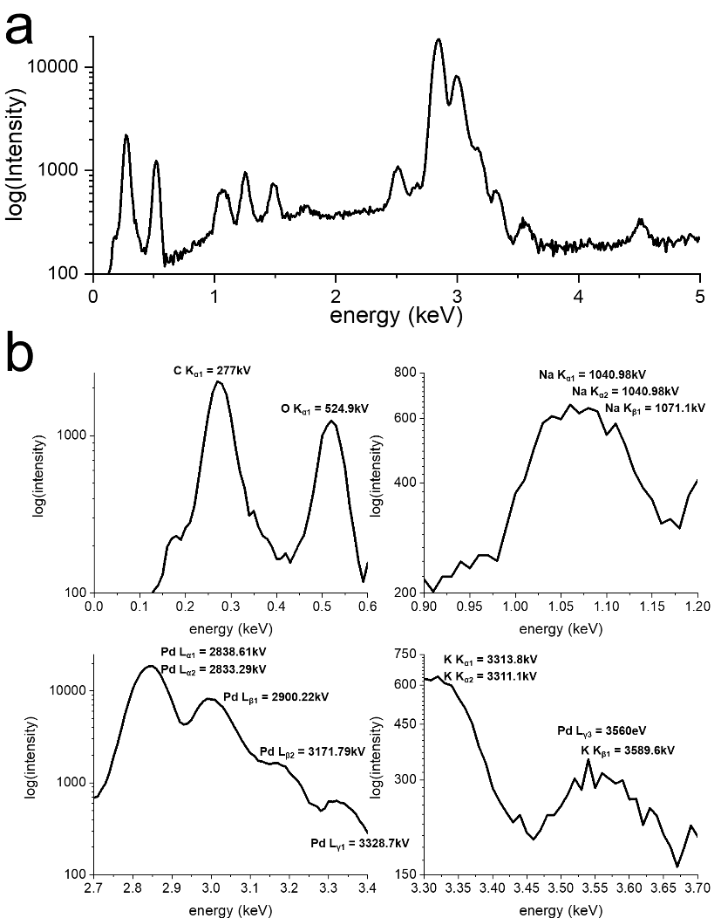

Figure. S1. (a) Overall energy-dispersive X-ray spectroscopy (EDS) spectrum of the as-synthesized Pd-40 acquired from scanning electron microscopy and (b) illustration of major peaks in four different ranges. Each characteristic X-ray peak signal is interpreted following ref.[1]. Along with strong signals of Pd L_{α}, low intensities of the characteristic X-ray peak for Na K_{α}, and K K_{β} are detected; however, due to the high background levels and peak signal overlaps, for instance between K K_{β} (3.57 keV) and Pd $L_{\gamma3}$ (3.56 keV)[1,2], acquiring good chemical resolution for the Pd nano-gels was difficult from X-ray based characterization.

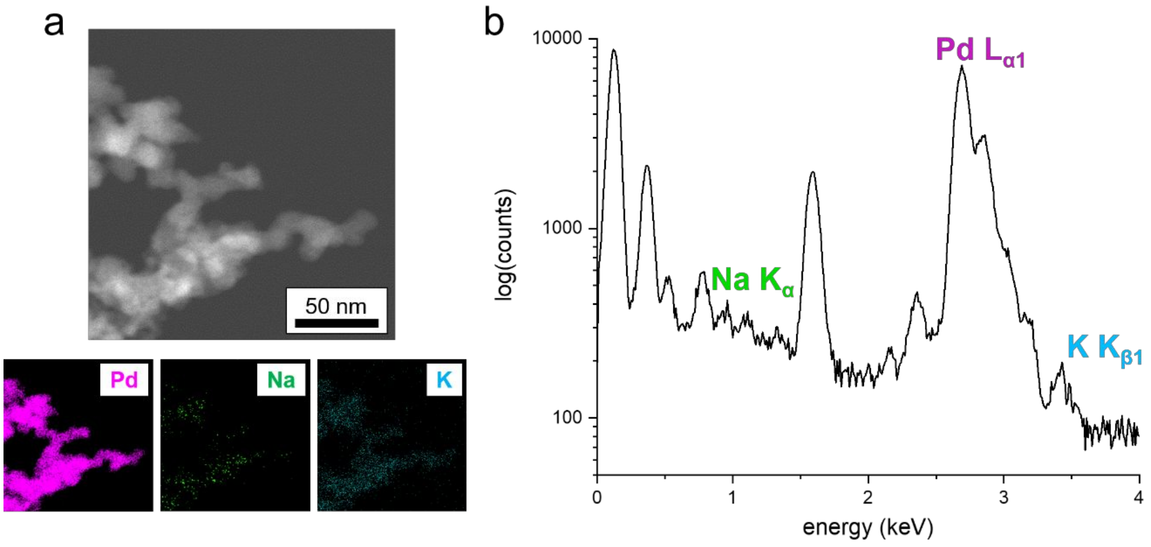

Figure. S2. (a) High-angle annular dark field (HAADF)- scanning transmission electron microscopy (STEM) images of the as-synthesized Pd-40 and corresponding EDS mapping of Pd (purple), Na (green), and K (cyan). (b) Overall EDS spectrum acquired from STEM.

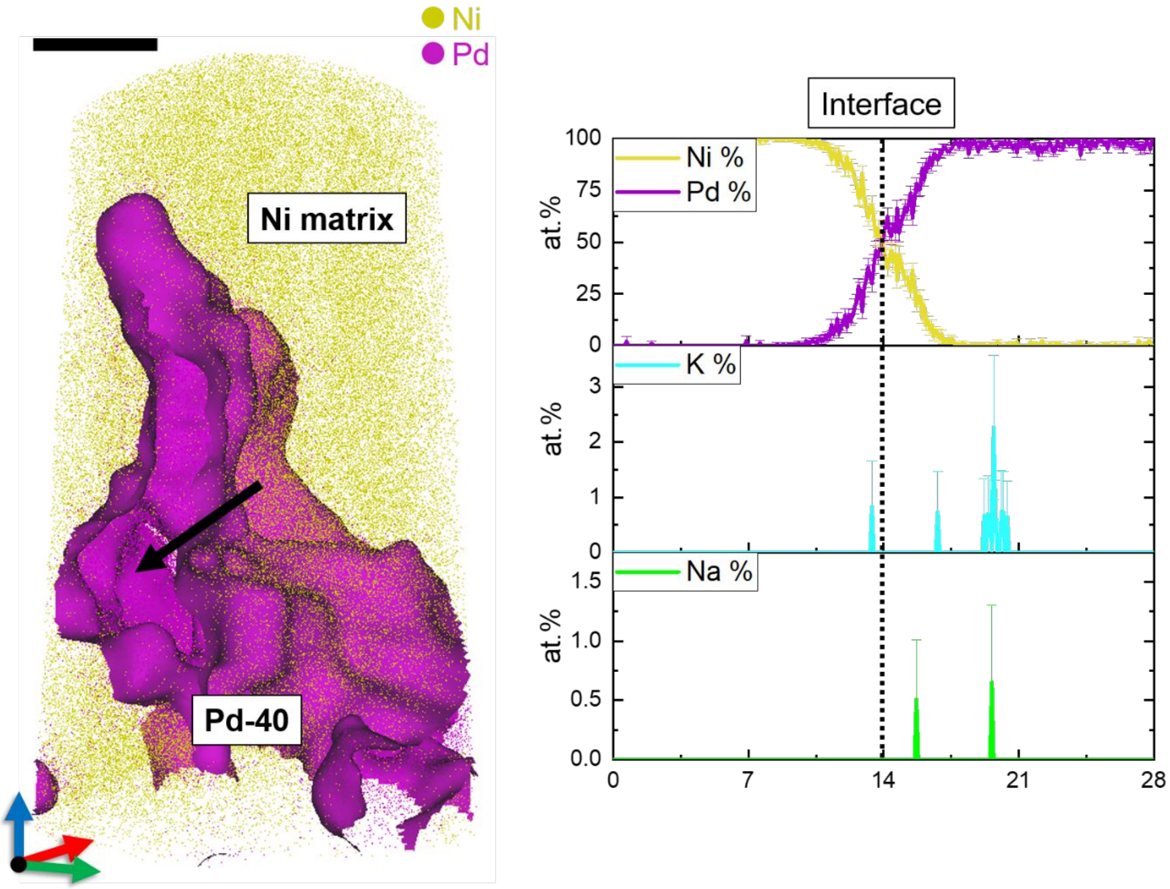

Figure. S3. 1D compositional profiles of the elements Ni, Pd, K, and Na contained in the Pd-40 nano-gels shown along the direction indicated by the black arrow in the 3D atom map on the left (scale bar 10 nm). The Pd/Ni interface is indicated by dotted line. A one-dimensional composition profile calculated within a cylindrical region of interest (Φ5 x 28 nm^3, 0.1 nm bin size) positioned perpendicular to the matrix-nanomaterial interface shows that most detected impurity element, i.e. Na, and K, are present inside the Pd nano-gels not on the surface.

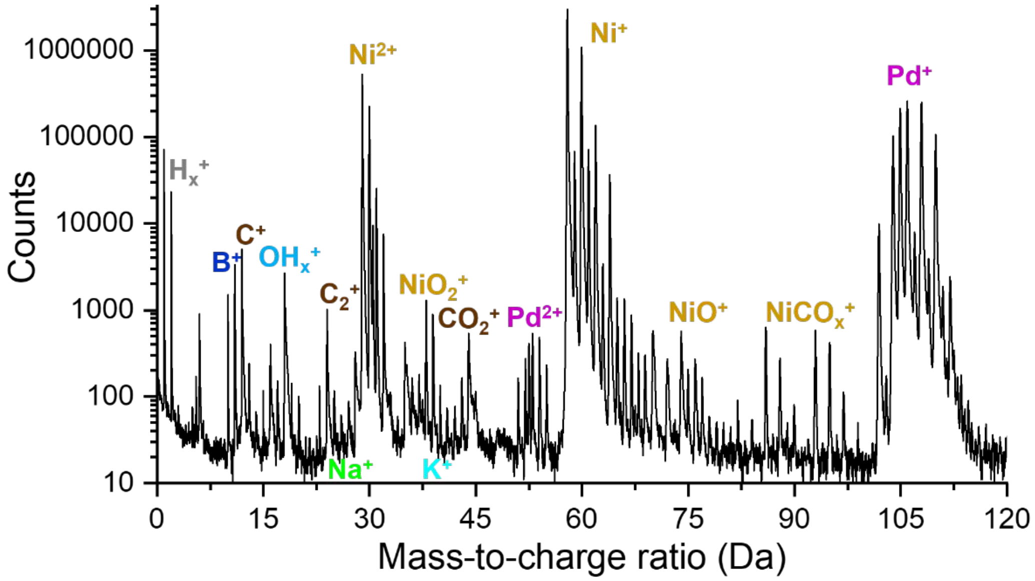

Figure. S4. Acquired overall mass spectrum of Pd nano-gels embedded in Ni matrix. C and O originate from H-citric acid ($C_6H_8O_7$) that was used as a buffer acid in co-electroplating sample preparation. Also B from sodium borohydride ($NaBH_4$) is detected but no segregation behavior at the grain boundaries (Figure. S11).

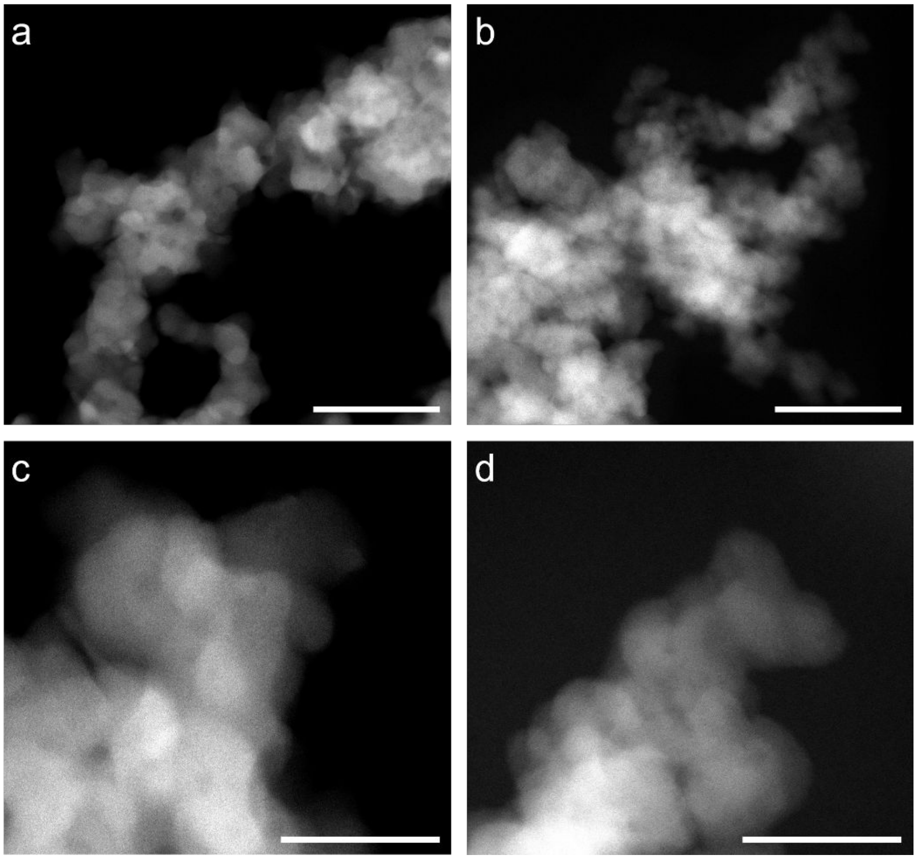

Figure. S5. HAADF-STEM images of (a,c) the Pd-40 and (b,d) the Pd-0.1 samples. Scale bars are 50 and 20 nm for (a,b) and (c,d), respectively.

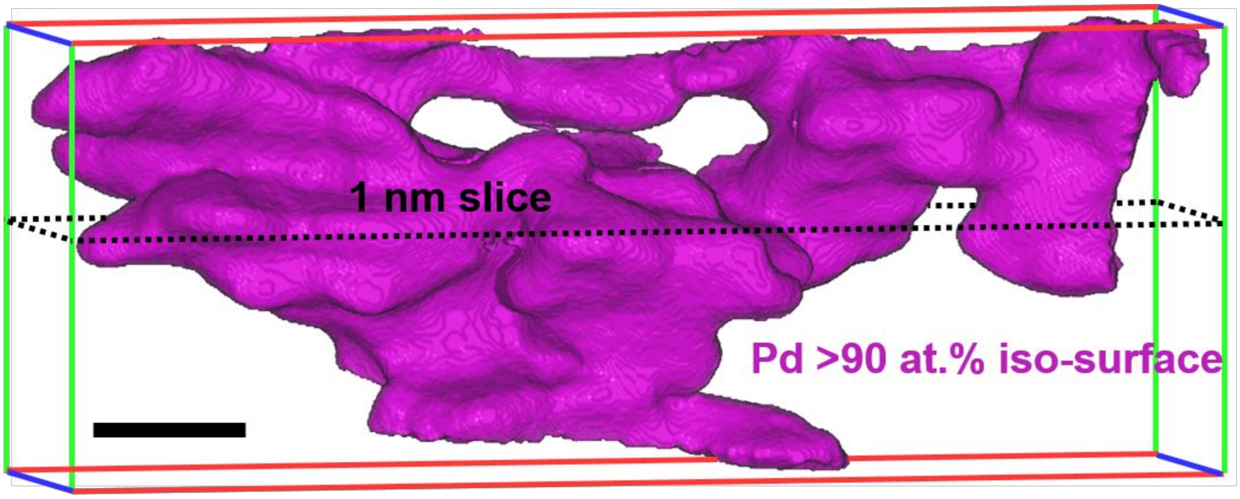

Figure. S6. 3D atom map of the Pd-0.1 sample. Scale bar is 20 nm. Tomogram of the 3D atom map is presented in Figure 2.

Table S1. Summary of the atomic composition analyses and comparison of Pd-40 and Pd-0.1 nanomaterials acquired from APT.

Element	Pd-40 (appm)	Pd-0.1 (appm)	ratio
Na	94 ±2	22 ±4	4.27
K	512 ±50	642 ±20	0.80

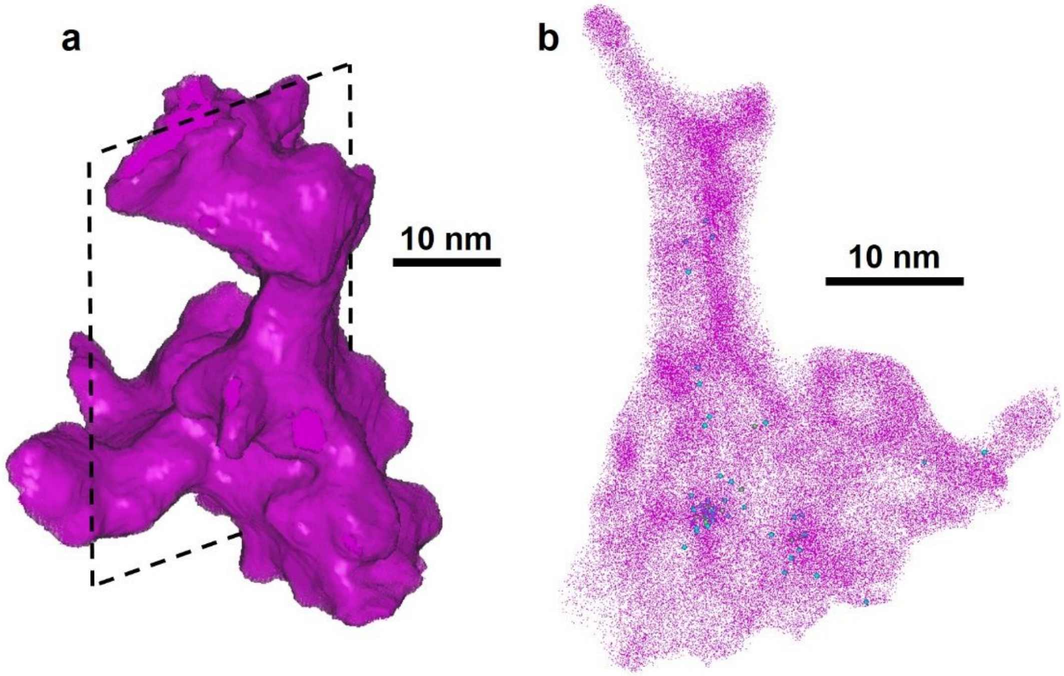

Figure S7. (a) 3D atom map of Pd-1 nano-gels. (b) 1-nm-thin sliced tomogram. Reconstructed Pd, Na, and K atoms are represented with purple, green, and cyan dots.

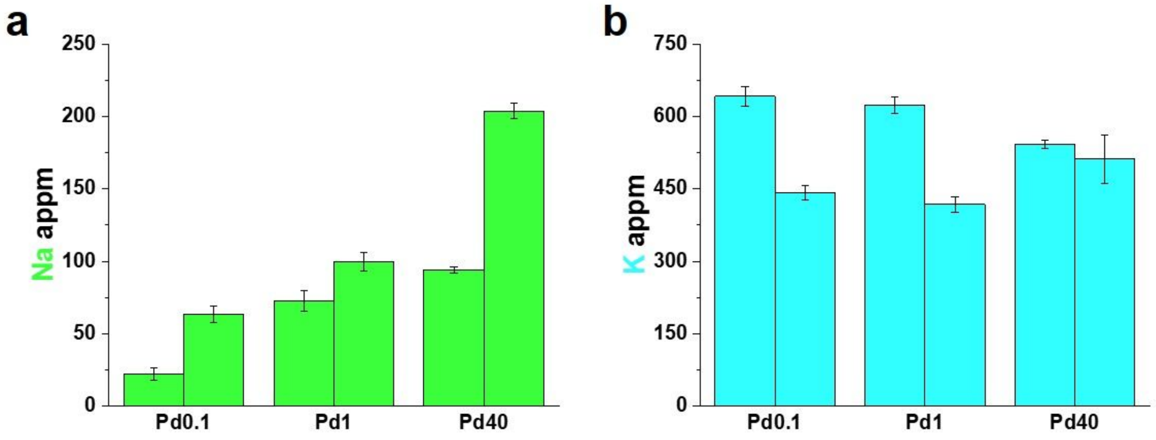

Figure S8. (a) Na and (b) K contents in each of the Pd gels.

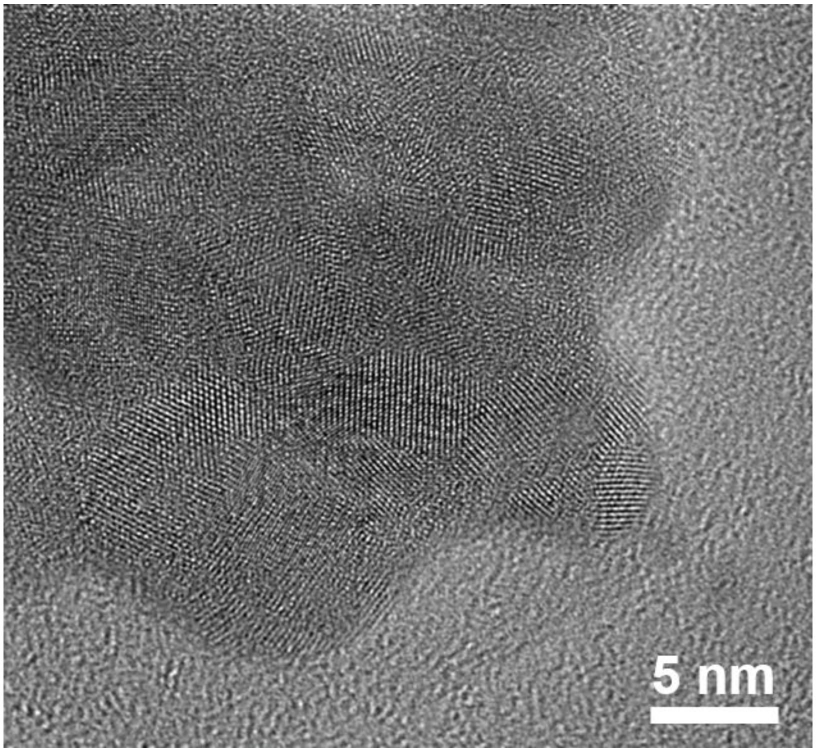

Figure. S9. High resolution TEM images of the Pd-0.1 sample.

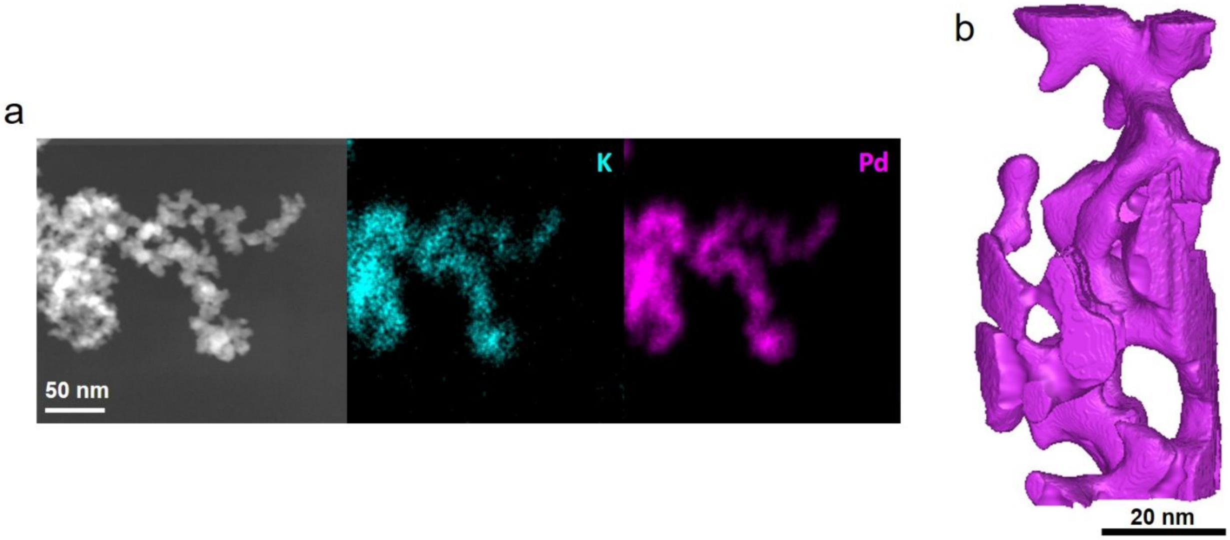

Figure S10. Synthesis of K-Pd-40 nano-gels to increase surface area-to-volume ratio by adding excess K during the synthesis. (a) STEM-EDS and (b) 3D atom map of the K-Pd-40 gels. KCl (>99.0%, Sigma Aldrich) was added at a 10 mole ratio level to the Pd precursor in a wet-chemical synthesis batch and followed by adding 40 mole ratio level of $NaBH_4$.

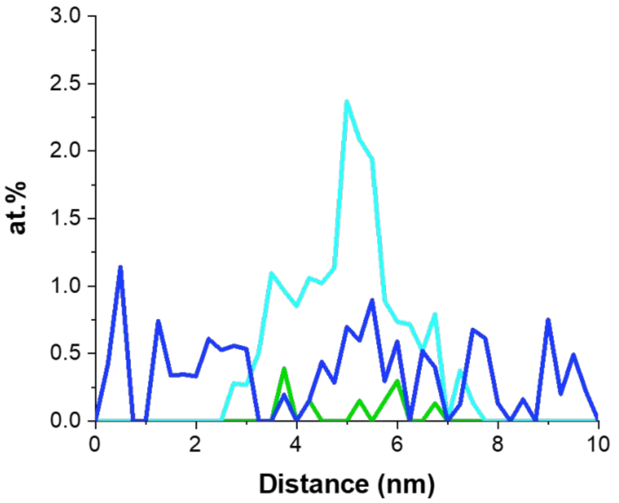

Figure. S11. 1D compositional profiles at the Pd-0.1 grain boundary of B (blue), Na (green), and K (cyan).

Density Functional Theory (DFT) Calculations

Surface adsorption calculations specific parameters

For the surface structures, a supercell containing a symmetric slab and 18 Å of vacuum is constructed. The Pd(111) slabs have 13 atomic layers (AL) and a thickness of 27.44 Å. The three outermost atomic layers are allowed to relax, while all other atoms are fixed at their bulk positions. The calculated surface energy of Pd(111) is 0.091 eV/Å^2, in good agreement with previous theoretical (0.082 meV/Å^2 [3], 0.099 meV/Å^2 [4]) and experimental (0.125 meV/Å^2 [5])work.

To account for the various coverages of alkalis on Pd(111) surface, differently sized surface unit cells are employed. The coverage (Θ), defined as the ratio between the number of adsorbate atoms and the number of Pd atoms in the surface layer, has the unit of monolayer (ML). For the binding sites, we consider the top, bridge, two 3-fold hollow (i.e. FCC and HCP) and the substitutional sites. In the sub-surface region, we considered the octahedral (OSS), tetrahedral (TSS), and substitutional (SSS) sites.

The binding energy (E_b) of alkali adsorbate with respect to their BCC phase is calculated as

$$E_\mathrm{b}(c_{X^q},T) = \frac{1}{2N_X}\left[E_\mathrm{tot}^{X:\mathrm{Pd\ surf}} - E_\mathrm{tot}^{\mathrm{Pd\ surf}} - 2N_X \cdot \mu_X(c_{X^q},T)\right]$$

where $E_\mathrm{tot}^{X:\mathrm{Pd\ surf}}$ and $E_\mathrm{tot}^{\mathrm{Pd\ surf}}$ are DFT calculated total energies of the alkali-substrate system, and the Pd substrate, respectively. N_X is the number of alkalis per surface cell. μ_X is the chemical potential of the alkali with respect to the BCC reference phases and accounting for the given experimental conditions, defined as

$$\mu_X(c_{X^q},T) = E_\mathrm{tot}^{X\ BCC} + \Delta\mu_X(c_{X^q}, T)$$

where $E_{\mathrm{tot}}^{X\,BCC}$ and c_{X^q} are DFT calculated total energy of the alkali bulk (*i.e.*, BCC Na, respectively K) and the concentration of alkali ion with a charge, q, in solution at a given temperature, T. $\Delta\mu_{\mathrm{X}}$ is the chemical potential shift for alkali ions (i.e., K^+ and Na^+) in solution with respect to the BCC reference phase calculated following ref.[6] as

$$\Delta\mu_X(c_{X^q}, T) = \Delta_f G^\circ(X^q) - \Delta_f G(X^q)(c_{X^q}, T) + q\mu_e(c_{X^q}, T)$$

where $\Delta_f G^\circ(X^q)$ is the formation energy of ion in the standard state with respect to the BCC bulk reference phase, which is derived by using tabulated literature data. $\Delta_f G(X^q)$ and μ_e are the formation energy of ion and the electron chemical potential at a given temperature and ion concentration. In the here discussed cases we use as input for the concentration of an alkali ion, c_{X^q}, the experimental values, to determine $-k_B T \ln\frac{c_{X^q}}{c_0}$ for $\Delta_f G(X^q)(c_{X^q}, T)$ where k_B and c_0 and the Boltzmann constant and the reference concentration c_0 = 55.55 mol/l (considering that 1l of water contains 55.55 mol of H_2O molecules). In addition, μ_e is determined utilizing the Nernst equation, which describes the change in the standard electrode potential due to changes in the concentration $q\mu_e(c_{X^q}, T) = qeU^\circ - k_{\mathrm{B}} T \ln\frac{c^\circ}{c_{X^q}}$. Here U° is the tabulated standard electrode potential at the reference concentration, c° = 1 mol/l. (for details, see Table S4)

Parameters specific to the grain boundary calculations

The relatively simple Σ5 (210) [100] symmetric tilt grain boundary (STGB) is selected as a representative high-angle GB in the present study. An inset in Figure. 3d shows the structure of the supercell used in all calculations. The supercell contains 40 atomic layers (4 atoms per layer, 160 atoms per cell) and represents a single cell doubled along the [100] and [012] directions. The slabs are large enough to avoid interactions between periodic images. Ionic relaxations were

allowed in all calculations within a fixed shape and volume. The free surface (FS) supercell is created by removing half the number of atoms within the cell located on one side of the GB. The supercell used for the bulk and FS model calculations have the exact same dimensions as that for the GB model. In all the calculations the alkali atoms replace one, respectively two, (*i.e.* Θ is 0.25, respectively 0.50) of four equivalent Pd atoms in the GB plane. All considered GB substitutional sites are indicated in the inset of Figure. 3d. The dimension of all the models was fixed during structural optimizations allowing relaxations only along the direction perpendicular to the GB plane. All structures have been rendered using the OVITO[7] program package and all GB structures created using the software described in ref.[8].

The impact alkali atoms have on stability of the GB is analyzed by the coalescence energy given by

$$E_{\mathrm{coa}}^{X} = \left[\left(E_{\mathrm{tot}}^{X:\mathrm{Pd\ FS}} - N_{\mathrm{Pd}}^{X:\mathrm{Pd\ FS}}E_{\mathrm{tot}}^{\mathrm{Pd\ bulk}} - N_{X}^{X:\mathrm{Pd\ FS}}E_{\mathrm{tot}}^{X\ \mathrm{BCC}}\right) - \left(E_{\mathrm{tot}}^{X:\mathrm{Pd\ GB}} - N_{\mathrm{Pd}}^{X:\mathrm{Pd\ GB}}E_{\mathrm{tot}}^{\mathrm{Pd\ bulk}} - N_{X}^{X:\mathrm{Pd\ GB}}E_{\mathrm{tot}}^{X\ \mathrm{BCC}}\right)\right]/A^{\mathrm{GB}}$$

where $E_{tot}^{X:\mathrm{Pd\ GB}}$ and $E_{tot}^{X:\mathrm{Pd\ FS}}$ are the total energies of the Pd GB and the Pd FS containing $N_{\mathrm{Pd}}^{X:\mathrm{Pd\ GB}}$ and $N_{\mathrm{Pd}}^{X:\mathrm{Pd\ FS}}$ Pd atoms and $N_{X}^{X:\mathrm{Pd\ GB}}$ and $N_{X}^{X:\mathrm{Pd\ FS}}$ alkalis corresponding to 0.0 ML, 0.25 ML and 0.50 ML in the GB plane, respectively. $E_{\mathrm{tot}}^{\mathrm{Pd\ bulk}}$ and $E_{\mathrm{tot}}^{\mathrm{X\ BCC}}$ are the total energies of the Pd FCC and the alkali BCC bulk phases. A^{GB} is the area of the GB plane in the supercell and it is same to the area of the FS plane.

Concentration analysis

The thermodynamic equilibrium concentrations of alkalis in the different Pd systems (*i.e.*, on Pd surface, in Pd bulk, and at Pd GB) are calculated in areal concentration unit (*i.e.*, cm^{-2}). For

comparison, the experimentally measured alkali contents in the MNAs are also converted to the areal concentrations for a comparison.

The upper-limit of the areal concentrations of alkalis integrated in MNAs for given experimental conditions are evaluated as

$$c^{\text{upper}-\text{limit}} = \frac{\Theta^{X:\text{Pd surf}}[E_{\text{b}}(c_{X^q},T) = 0]}{A^{\text{Pd surf}}}$$

where $\Theta^{X:Pd\,surf}[E_{\text{b}}(c_{X^q},T) = 0]$ and $A^{\text{Pd surf}}$ are the maximum coverage, determined as the value at which the binding energy of an alkali with respect to the ion in solution becomes zero at the given conditions, and the area of the Pd(111) $p(1\times1)$ surface unit cell, respectively.

The areal concentrations of alkalis in Pd bulk and in Pd GB plane are evaluated based on the Boltzmann distribution as follows:

$$c^{X:\alpha} = \frac{N_{\text{site}} \cdot e^{\frac{-E_f^{X:\alpha}(c_{X^q},T)}{k_{\text{B}}T}}}{A^{\alpha}}$$

where α is the considered Pd systems [*i.e.* a Pd (4×4×4) cubic supercell with 256 Pd atoms or the Pd GB supercell with 160 Pd atoms]. N_{site} and A^{α} are the number of sites in which the alkalis can be substituted (*i.e.*, 256 for the Pd cubic bulk cell and 4 in the Pd GB plane) and the area of the corresponding system, respectively. $E_f^{X:\alpha}(c_{X^q},T)$ is the formation energies of an alkali atom in the corresponding system at the given condition calculated as

$$E_f^{X:\alpha}(c_{X^q},T) = E_{\text{tot}}^{X:\alpha} - E_{\text{tot}}^{\alpha} - [E_{\text{tot}}^{X} + \Delta\mu_X(c_{X^q}, T)]$$

where E_{tot}^{α}, $E_{\text{tot}}^{X:\alpha}$, and E_{tot}^{X} are the total energies of the pure Pd system, α, the corresponding Pd systems with a single substitutional alkali atom, and the alkali BCC bulk reference, respectively. Δ

$\mu_X(c_{X^q}, T)$ is the chemical potential shift for alkali ions in solution with respect to the BCC reference phase at the given conditions.

The areal concentrations of experimentally measured alkali contents in MNAs are evaluated based on the assumption that alkali atoms are present only at a single GB plane in the Pd cubic cell as follows:

$$c^{X:\mathrm{APT}} = \frac{N^{X:\mathrm{MNA}}(c_{X^q}, T)}{A^{\mathrm{cubic\ Pd}}}$$

where $N^{X:\mathrm{MNA}}(c_{X^q}, T)$ is the experimentally measured alkali contents in the MNAs at the given conditions in an appm unit and $A^{\mathrm{cubic\ Pd}}$ is an area of the assumed GB plane in the cubic Pd cell containing a milion Pd atoms. Detailed data for the concentration calculations are listed in Table S7.

Table S2. Calculated binding energies for Na adsorbed on Pd(111) with respect to the Na BCC bulk phase for several Na coverages Θ and different adsorption sites.

E_b (eV/Na atom)						
**Θ (ML)**	**1.00**	**0.75**	**0.50**	**0.25**	**0.11**	**0.06**
**Top**	**0.119**	**-0.150**	**-**	**-0.888**	**-**	**-**
**FCC**	**-0.091**	**-0.366**	**-0.624**	**-1.047**	**-1.328**	**-1.470**
**HCP**	**-0.094**	**-0.340**	**-0.626**	**-1.047**	**-1.333**	**-**
**Bridge**	**-0.061**	**-0.360**	**-**	**-1.028**	**-**	**-**
**TetraI sub-surf**	**-0.028**	**-0.026**	**-**	**2.183**	**-**	**-**
**Octa sub-surf**	**-0.205**	**-0.154**	**0.214**	**2.513**	**-**	**-**
**Substitutional**	**-0.101**	**-0.421**	**-0.697**	**-0.977**	**-0.947**	**-1.050**
**Subst sub-surf**	**-0.215**	**-0.339**	**-0.418**	**-0.476**	**-0.325**	**-**

Table S3. Calculated binding energies for K absorbed on Pd(111) with respect to the K BCC bulk phase for several K coverages Θ and different adsorption sites.

E_b (eV/K atom)						
**Θ (ML)**	**1.00**	**0.75**	**0.50**	**0.25**	**0.11**	**0.06**
**Top**	**1.611**	**0.649**	**-0.248**	**-1.237**	**-**	**-**
**FCC**	**1.405**	**0.544**	**-0.336**	**-1.286**	**-1.664**	**-1.922**
**HCP**	**1.393**	**0.536**	**-0.335**	**-1.290**	**-1.708**	**-**
**Bridge**	**1.408**	**-0.250**	**-0.290**	**-1.283**	**-**	**-**
**TetraI sub-surf**	**1.210**	**0.681**	**-**	**1.852**	**-**	**-**
**Octa sub-surf**	**1.065**	**0.609**	**0.462**	**1.836**	**-**	**-**
**Substitutional**	**1.395**	**0.564**	**-0.155**	**-0.993**	**-1.093**	**-1.286**
**Subst sub-surf**	**1.058**	**0.873**	**0.921**	**1.539**	**1.842**	**-**

Table S4. Tabulated cohesive energies (E_{coh}), standard (T° = 298.15K, p° = 1 bar) ionization energies ($\Delta_{\mathrm{IE}}G^{\circ}$), ion-hydration energies ($\Delta_{\mathrm{hyd}}G^{\circ}$), and standard reduction potentials (eU°) taken from Refs. [9,10]. The standard Gibbs free energies of formation ($\Delta_f G^{\circ}$) is calculated as $\Delta_f G^{\circ} = E_{\mathrm{coh}} + \Delta_{\mathrm{IE}}G^{\circ} + \Delta_{\mathrm{hyd}}G^{\circ}$ based on Ref. [6]. The Gibbs free energy of formation ($\Delta_f G$) of ion (Na^+ or K^+) and the electron chemical potential (μ_e) calculated for the chemical potential shift ($\Delta\mu_i$) of element i (N or K) corresponding to the experimental conditions with respect to the BCC bulk phase are also listed.

**Tabulated standard energies (in eV)**						
**Element**	E_{coh}[9]	$\Delta_{\mathrm{IE}}G^{\circ}$[9]	$\Delta_{\mathrm{hyd}}G^{\circ}$[10]	$\Delta_f G^{\circ}$	eU°[9]	-
**Na**	**1.114**	**5.139**	**-3.782**	**2.471**	**-2.71**	**-**
**K**	**0.922**	**4.341**	**-3.057**	**2.206**	**-2.931**	**-**
**$\Delta_f G$, μ_e and $\Delta\mu_i$ at the given condition (in eV)**						
**Ion**	$\Delta_f G$ (Pd-40)	μ_e(Pd-40)	$\Delta\mu_X$ (Pd-40)	$\Delta_f G$ (Pd-0.1)	μ_e (Pd-0.1)	$\Delta\mu_X$ (Pd-0.1)
**Na+**	**0.128**	**-2.734**	**-0.390**	**0.282**	**-2.889**	**-0.700**
**K+**	**0.205**	**-3.032**	**-1.031**	**0.205**	**-3.032**	**-1.031**

Table S5. Calculated binding energies for Na adsorbed on Pd(111) with respect to a Na^+ ion in solution at the given experimental condition for several Na coverages Θ and different adsorption sites.

E_b (Pd-40) (eV/Na atom)						
**Θ (ML)**	**1.00**	**0.75**	**0.50**	**0.25**	**0.11**	**0.06**
**Top**	**0.509**	**0.240**	**-**	**-0.498**	**-**	**-**
**FCC**	**0.299**	**0.054**	**-0.234**	**-0.657**	**-0.938**	**-1.080**
**HCP**	**0.296**	**0.050**	**-0.236**	**-0.657**	**-0.943**	**-**
**Bridge**	**0.329**	**0.030**	**-**	**-0.638**	**-**	**-**
**TetraI sub-surf**	**0.362**	**0.364**	**-**	**2.573**	**-**	**-**
**Octa sub-surf**	**0.185**	**0.236**	**0.604**	**2.903**	**-**	**-**
**Substitutional**	**0.289**	**-0.031**	**-0.307**	**-0.587**	**-0.557**	**-0.660**
**Subst sub-surf**	**0.175**	**0.051**	**-0.028**	**-0.086**	**0.065**	**-**
**E_b (Pd-0.1) (eV/Na atom)**						
**Θ (ML)**	**1.00**	**0.75**	**0.50**	**0.25**	**0.11**	**0.06**
**Top**	**0.819**	**0.550**	**-**	**-0.188**	**-**	**-**
**FCC**	**0.609**	**0.364**	**0.076**	**-0.347**	**-0.628**	**-0.770**
**HCP**	**0.606**	**0.360**	**0.074**	**-0.347**	**-0.633**	**-**
**Bridge**	**0.639**	**0.340**	**-**	**-0.328**	**-**	**-**
**TetraI sub-surf**	**0.672**	**0.674**	**-**	**2.883**	**-**	**-**
**Octa sub-surf**	**0.495**	**0.546**	**0.914**	**3.213**	**-**	**-**
**Substitutional**	**0.599**	**0.279**	**0.003**	**-0.277**	**-0.247**	**-0.350**
**Subst sub-surf**	**0.485**	**0.361**	**0.282**	**0.224**	**0.375**	**-**

Table S6. Calculated binding energies for K adsorbed on Pd(111) with respect to a K^+ ion in solution at the given experimental condition for several K coverages Θ and different adsorption sites.

E_b (Pd-40 = Pd-0.1) (eV/K atom)						
**Θ (ML)**	**1.00**	**0.75**	**0.50**	**0.25**	**0.11**	**0.06**
**Top**	**2.642**	**1.680**	**0.783**	**-0.206**	**-**	**-**
**FCC**	**2.436**	**1.575**	**0.695**	**-0.255**	**-0.633**	**-0.891**
**HCP**	**2.424**	**1.567**	**0.696**	**-0.259**	**-0.677**	**-**
**Bridge**	**2.439**	**0.781**	**0.741**	**-0.252**	**-**	**-**
**TetraI sub-surf**	**2.241**	**1.712**	**-**	**2.883**	**-**	**-**
**Octa sub-surf**	**2.096**	**1.640**	**1.493**	**2.867**	**-**	**-**
**Substitutional**	**2.426**	**1.595**	**0.876**	**0.038**	**-0.062**	**-0.255**
**Subst sub-surf**	**2.089**	**1.904**	**1.952**	**2.570**	**2.873**	**-**

Table S7. Data for the concentration analysis shown in Figure. 3 of the main text at the given systems and at the given experimental conditions.

**Pd surface**			
**Condition**	$\Theta^{X-\mathrm{S}}$ **(ML)**	$A^{\mathrm{Pd\ surf}}$**(cm²)**	$c^{\mathrm{upper\ limit}}$
**Na-Pd40**	**0.74**	**6.79×10⁻¹⁶**	**1.08×10¹⁵**
**Na-Pd0.1**	**0.52**		**7.72×10¹⁴**
**K-Pd40**	**0.31**		**4.51×10¹⁴**
**K-Pd0.1**	**0.31**		**4.51×10¹⁴**
**Pd bulk**			
**Condition**	$E_f^{X\mathrm{:Pd\ bulk}}$ **(eV/atom)**	$A^{\mathrm{Pd\ bulk}}$**(cm²)**	$c^{X\mathrm{:Pd\ bulk}}$
**Na-Pd40**	**0.279**	**2.50×10⁻¹⁴**	**2.12×10¹¹**
**Na-Pd0.1**	**0.589**		**1.33×10⁶**
**K-Pd40**	**2.735**		**1.15×10⁻³⁰**
**K-Pd0.1**	**2.735**		**1.15×10⁻³⁰**
**Pd GB**			
**Condition**	$E_f^{X\mathrm{:Pd\ GB}}$ **(eV/atom)**	$A^{\mathrm{Pd\ GB}}$**(cm²)**	$c^{X\mathrm{:Pd\ GB}}$
**Na-Pd40**	**-0.194**	**6.86×10⁻¹⁵**	**1.46×10¹⁴**
**Na-Pd0.1**	**0.115**		**6.73×10¹²**
**K-Pd40**	**1.556**		**4.21×10⁻¹²**
**K-Pd0.1**	**1.556**		**4.21×10⁻¹²**
**Pd MNA**			
**Condition**	$N^{X\mathrm{:MNA}}$ **(appm)**	$A^{\mathrm{cubic\ Pd}}$**(cm²)**	$c^{X\mathrm{:APT}}$
**Na-Pd40**	**94**	**6.22×10⁻¹²**	**1.51×10¹³**
**Na-Pd0.1**	**22**		**3.54×10¹²**
**K-Pd40**	**512**		**8.23×10¹³**
**K-Pd0.1**	**642**		**1.03×10¹⁴**

References

(1) Thompson; Thompson, A.; Attwood, D.; Gullikson, E.; Howells, M.; Kim, K.-J.; Kirz, J.; Kortright, J.; Lindau, I.; Liu, Y.; et al. X-Ray Data Booklet. *Lawrence Berkeley National Laboratory*. 2009, p 176.

(2) Sultanova, E. D.; Samigullina, A. I.; Nastapova, N. V; Nizameev, I. R.; Kholin, K. V; Morozov, V. I.; Gubaidullin, A. T.; Yanilkin, V. V; Kadirov, M. K.; Ziganshina, A. Y.; et al. Highly Active Pd–Ni Nanocatalysts Supported on Multicharged Polymer Matrix. *Catal. Sci. Technol.* **2017**, *7* (24), 5914–5919. https://doi.org/10.1039/C7CY01797A.

(3) Da Silva, J. L. F.; Stampfl, C.; Scheffler, M. Converged Properties of Clean Metal Surfaces by All-Electron First-Principles Calculations. *Surf. Sci.* **2006**, *600* (3), 703–715. https://doi.org/https://doi.org/10.1016/j.susc.2005.12.008.

(4) Yoo, S. H.; Lee, J. H.; Delley, B.; Soon, A. Why Does Bromine Square Palladium off? An Ab Initio Study of Brominated Palladium and Its Nanomorphology. *Phys. Chem. Chem. Phys.* **2014**, *16* (34), 18570–18577. https://doi.org/10.1039/c4cp02384f.

(5) Tyson, W. R.; Miller, W. A. Surface Free Energies of Solid Metals: Estimation from Liquid Surface Tension Measurements. *Surf. Sci.* **1977**, *62*, 267.

(6) Todorova, M.; Neugebauer, J. Extending the Concept of Defect Chemistry from Semiconductor Physics to Electrochemistry. *Phys. Rev. Appl.* **2014**, *1*, 14001.

(7) Stukowski, A. Visualization and Analysis of Atomistic Simulation Data with OVITO–the Open Visualization Tool. *Model. Simul. Mater. Sci. Eng.* **2009**, *18* (1), 15012. https://doi.org/10.1088/0965-0393/18/1/015012.

(8) Hadian, R.; Grabowski, B.; Neugebauer, J. GB Code: A Grain Boundary Generation Code. *J. Open Source Softw.* **2018**, *3* (29), 900. https://doi.org/10.21105/joss.00900.

(9) Lide, D. R. *CRC Handbook of Chemistry and Physics 86th Ed.*; CRC Press, Boca Raton, FL, 2005.

(10) Marcus, Y. The Thermodynamics of Solvation of Ions. Part 2.—The Enthalpy of Hydration at 298.15 K. *J. Chem. Soc., Faraday Trans. 1* **1987**, *83*, 339.
